# Assay validation and clinical performance of chronic inflammatory and chemokine biomarkers of NASH fibrosis

**DOI:** 10.1371/journal.pone.0217263

**Published:** 2019-07-10

**Authors:** Sumit Kar, Sabina Paglialunga, Sharon H. Jaycox, Rafiqul Islam, Angelo H. Paredes

**Affiliations:** 1 Bioanalytical Services, Celerion, Lincoln, NE, United States of America; 2 Scientific Affairs, Celerion, Tempe, AZ, United States of America; 3 Early Clinical Research, Celerion, Tempe, AZ, United States of America; 4 Internal Medicine, Gastroenterology and Hepatology Services, Brooke Army Medical Center, San Antonio, TX, United States of America; University of Southern California, UNITED STATES

## Abstract

Nonalcoholic steatohepatitis (NASH) is a chronic liver disease that can lead to cirrhosis, liver transplant, and even hepatocellular carcinoma. While liver biopsy remains the reference standard for disease diagnosis, analytical and clinical development of non-invasive soluble biomarkers of NASH are of great importance to advance the field. To this end, we performed analytical and clinical validation on a series of pro-inflammatory cytokines and chemokines implicated hepatic inflammation; IL-6, CRP, TNFα, MCP-1, MIP-1β, eotaxin, VCAM-1. Biomarker assays were validated for accuracy and precision. Clinical performance was evaluated in a random sample of 52 patients with biopsy-proven NAFLD/NASH. Patients were categorized into three groups according to their fibrosis stage; advanced (F3-F4), mild (F1-2) and no (F0) fibrosis. Serum IL-6 was increased in patients with advanced fibrosis (2.71 pg/mL; 1.26 pg/mL; 1.39 pg/mL p<0.01) compared to patients with mild or no fibrosis respectively. While, there was no significant difference noted in CRP, TNFα, MCP-1, MIP-1β, eotaxin among the three groups, VCAM-1 levels were increased by 55% (p<0.01) and 40% (p<0.05) in the advanced cohort compared to the mild and no fibrosis groups respectively. VCAM-1 also displayed good clinical performance as a biomarker of advanced fibrosis with an area under the receiver operating curve of 0.87. The VCAM-1 assay demonstrated robust accuracy and precision, and VCAM-1 outperformed IL-6, CRP, TNFα, and the chemokines MCP-1, MIP-1β, and eotaxin as a biomarker of advanced fibrosis in NASH. Addition of biomarkers such as IL-6 and VCAM-1 to panels may yield increased sensitivity and specificity for staging of NASH.

## Introduction

Nonalcoholic steatohepatitis (NASH) is a chronic metabolic liver disorder which can progress to hepatic fibrosis, cirrhosis, end-stage liver disease, and hepatocellular carcinoma. Recent estimates indicate that the prevalence of NASH in the general adult population is 3–5% [1], making NASH the second leading etiology for a liver transplant [2]. Moreover, increased prevalence of underlying risk factors such as obesity and type 2 diabetes, along with greater recognition of the disease and diagnosis by primary care physicians [3], are anticipated to greatly impact the NASH diagnosis rates over the next several years. In this regard, by 2030 the number of NASH cases with advanced fibrosis is anticipated to be nearly 8 million in the US, an increase of over 160% from recent estimates [4].

Currently, the diagnosis and prognosis of NASH, as well as assessment of treatment response, are based on liver biopsy results. A major criticism of this approach is that liver biopsies are invasive procedures, which can be painful and associated with severe complications. Although considered the reference standard, liver biopsies are afflicted by sampling error, inter-operational variability, as well as low patient acceptance rates (reviewed in [5]). While fibrosis panels based on clinical chemistry laboratory results such as FIB4 are simple tools for discriminating between disease stages, its diagnostic performance is inadequate [6]. Therefore, non-invasive soluble biomarkers with robust analytical and clinical validation for diagnosing NASH and fibrosis severity are required to overcome these challenges.

Accumulation of toxic lipid species within the liver tissue associated with metabolic disturbances, insulin resistance, and inflammation can lead to hepatocellular stress, injury and cell death resulting in hepatic stellate cell (HSC) activation and upregulation of fibrogenesis. Although fibrosis is not a component of the NASH diagnosis [7, 8], fibrosis is the main contributing factor to liver-related mortality in NASH patients [9]. Hepatic inflammation is a hallmark of the disease and plays a key role in the pathophysiology driving fibrosis progression. Inflammatory cytokines such as interleukin-6 (IL-6), C-reactive protein (CRP) and tumor necrosis factor alpha (TNFα) as well as chemokines orchestrate the upregulation of resident Kupffer cells, activation of HSC and infiltration of immune cells [10–12]. Macrophage recruitment is regulated by macrophage chemoattractant protein 1 (MCP1/CCL2), macrophage inflammation protein 1β (MIP-1β/CCL4) and eotaxin (CCL11). Other immune cells such as lymphocytes can be recruited by vascular cell adhesion molecule 1 (VCAM-1) (reviewed in [13]). Some of these chemokines, particularly MIP-1β and eotaxin, have never been studied in NASH fibrosis. While VCAM-1 is classically known for its role in endothelial dysfunction and angiogenesis, it is interesting to note that VCAM-1 expression is also upregulated during chronic inflammation not only on the endothelial cell surface but also in macrophages, dendritic cells and Kupffer cells in the liver [14].

To qualify use of biomarkers in a pharmacological clinical trial and regulatory submission, the Food and Drug Administration’s (FDA) Center for Drug Evaluation and Research (CDER) developed the Biomarker Qualification Program to establish standards for validating the analytical measurement and clinical utility of a biomarker for a specific context of use (COU) [15]. Validation of both the analytical measurement and the performance of the biomarker for its intended COU must be established for a specific disease like NASH. Furthermore, FDA Bioanalytical Guidelines and the Critical Path Institute establish standards for validating a biomarker assay [16, 17]. Both documents detail biomarker assay requirements for a given COU such as an exploratory or secondary endpoint in a clinical trial.

Although several of these cytokines have been evaluated in previous biomarker studies of NASH [18, 19], no single study has evaluated and compared a large set of cytokines and chemokines for their potential as a biomarker of NASH fibrosis where all subjects were diagnosed by liver biopsy with assays that underwent robust analytical validation. In this study, we performed analytical assay validation on a set of seven pro-inflammatory cytokine and macrophage recruitment chemokine biomarkers according to these guidelines and clinically examined their performance as a biomarker of fibrosis severity in biopsy-confirmed NASH patients.

## Material and methods

The test population were subjects enrolled in an on-going tissue and serum repository at Brooke Army Medical Center, TX. The study protocol was approved by the Brooke Army Medical Center ethics review board and conducted according to the principles of the Declaration of Helsinki. All subjects gave their written informed consent to participate in the study. All subjects underwent a liver biopsy due to the clinical suspicion of NASH. NAFLD was defined by consumption of less than 20 and 30 grams of alcohol every day for women and men, respectively and a liver biopsy showing fatty liver disease [20]. Histology was assessed by a single hepatobiliary pathologist using the NASH-Clinical Research Network criteria [7]. The presence of steatohepatitis was defined by steatosis, inflammation and cytologic ballooning. NASH severity was categorized as liver grade ranging between 0 and 2. Participants were categorized based on hepatic fibrosis stage; no fibrosis (F0), mild fibrosis (F1-F2) and advanced fibrosis (F3-F4). Twelve-hour fasting serum was obtained from all subjects the day of their liver biopsy and processed for clinical laboratory evaluations including liver function tests, glucose, insulin, and lipids. An aliquot of serum was stored at -80°C for biomarker analysis. FIB-4 was calculated as previously reported [6].

### Biomarker measurement and analytical validation

A random series sample of NAFLD/NASH patient serum from the Brooke Army Medical Center repository was used in the current study. The serum levels of IL-6 and TNF-α were measured using the human V-plex Proinflammatory Panel 2 (MSD, Kenilworth, NJ). MCP-1, MIP-1β, and eotaxin were measured using the human V-plex Chemokine Panel 1 (MSD). The serum levels of VCAM-1 and CRP were measured using the human V-plex Vascular Injury Panel 2 (MSD). All assays were modified appropriately to meet the FDA bioanalytical guidance and industry best practices. For all assays, three quality control samples were prepared in-house by spiking recombinant protein in an appropriate surrogate matrix to span the entire standard curve range. These quality controls (QCs) were utilized in addition to kit QCs to verify assay performance. The cytokine concentrations were determined from the standard curve with at least six points using a 4-parameter logistic fit curve to transform the mean light intensities into concentrations using MesoScale Discovery Workbench 4.0 (Rockville, MD).

Assays were validated for accuracy and precision before sample analysis. The dynamic range for each analyte was determined by the lower limit of quantification (LLOQ) and the upper limit of quantification (ULOQ) as the lowest and highest quantifiable standard, respectively, within 25% of the nominal value. Assay accuracy and precision were established by quantifying 6 QCs along the standard curve range including an endogenous QC in human serum QCs at the LLOQ and ULOQ in 2–3 independent batches run on different days. A calculated concentration <±20% of the nominal value for the QCs and <±25% for the LLOQ and ULOQ QCs was set for acceptable accuracy and a coefficient of variation (%CV) <±20% the QCs and <±25% for the LLOQ and ULOQ QCs was set for acceptable precision. Analytical assay validation results are presented as Bland-Altman plots, with the average cytokine concentration plotted against the percent difference between the nominal and measured values.

### Statistical analysis

Results are expressed as mean±SD. Statistically significant differences were evaluated by a one-way ANOVA followed by Tukey’s post-hoc test or Chi-squared analysis where appropriate. Associations among variables were determined using the Pearson coefficient (R). Biomarker performance to distinguish differences among cohorts was analyzed by the area-under-the ROC (AUROC) with 95% confidence intervals (CI), compared to biopsy results. Statistical significance was set at p<0.05. Graphs and statistical analyses were performed using GraphPad Prism 7 (GraphPad Software, La Jolla, CA).

## Results

### Assay validation

For a biomarker assay to be used for diagnostic or drug development purposes, at minimum, precision and accuracy of the assay should be verified [21, 22]. The dynamic range of the biomarker assays were determined to be 2.45 pg/mL– 444 pg/mL for IL-6; 69.2 pg/mL– 108,000 pg/mL for CRP; 0.79 pg/mL– 211 pg/mL for TNFα; 8.28 pg/mL—1060 pg/mL for MCP-1, 17.5 pg/mL– 2240 pg/mL for MIP-1β; 56.8 pg/mL– 3640 pg/mL for eotaxin;—and 90.8 pg/mL- 284,000 pg/mL for VCAM-1. The endogenous concentrations of analytes in serum for all subjects were within these analytical ranges.

**Fig 1:**
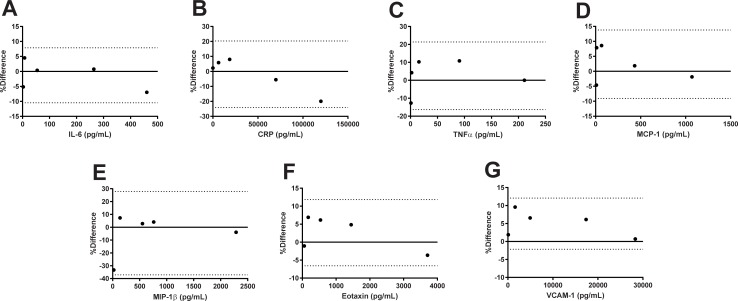
Assay validation Bland-Altman plots. (A) IL-6, (B) CRP, (C) TNFα, (D) MCP-1, (E) MIP-1β, (F) Eotaxin, (G) VCAM-1. Dotted lined indicates 95% limits of agreement.

Precision and accuracy of each assay met FDA bioanalytical guidelines: accuracy (% Theoretical) was determined to be between 80% and 120% of the nominal values for the QCs for each assay and precision (% Bias) was determined to be <20% for each assay (Table 1). In addition, Bland-Altman plots revealed the percent difference between the nominal value and measured mean for each QC is within the 95% limits of agreement (Fig 1).

**Table 1:** Assay validation precision and accuracy.

Biomarker	Nominal Concentration (pg/mL)	Mean Measured Concentration (pg/mL)	% CV	% Theoretical
IL-6	2.45 (LLOQ)	2.58	1.0	105.3
	7.72	7.38	3.3	95.6
	54.50	54.30	3.2	99.6
	265	263	0.9	99.2
	444 (ULOQ)	476	2.2	107.2
CRP	69.2 (LLOQ)	67.7	1.4	97.8
	6440	6073	0.5	94.3
	1940	17907	1.2	92.3
	68000	71871	0.2	105.7
	108000 (ULOQ)	131880	4.0	122.1
TNFα	0.79 (LLOQ)	0.87	3.0	113.5
	2.17	2.08	4.2	95.9
	16.4	14.8	1.4	90.2
	95.5	85.7	2.9	89.7
	211 (ULOQ)	211	2.5	100.0
MCP-1	8.28 (LLOQ)	8.67	1.8	104.8
	11.9	11.0	9.8	92.4
	69.2	63.5	5.5	91.8
	438	430	3.2	98.2
	1060 (ULOQ)	1080	4.3	99.5
MIP-1β	17.5 (LLOQ)	24.5	7.8	90.4
	142	132.0	4.6	93.0
	560	544.5	1.9	97.2
	776	745	1.3	96.0
	2240 (ULOQ)	2330	0.4	104.0
Eotaxin	56.8 (LLOQ)	57.4	4.8	101.0
	180	168.0	2.3	93.3
	553	520	4.5	94
	1480	1410	2.2	95.3
	3640 (ULOQ)	3775	8.6	103.7
VCAM-1	90.8 (LLOQ)	89.1	1.0	98.1
	1690	1536	0.6	90.9
	5110	4786	0.9	93.7
	17900	16826	1.8	94.1
	28400 (ULOQ)	28201	5.1	99.3

CV; coefficient of variance; LLOQ; lower limit of quantification, ULOQ; upper limit of quantification

### NASH patient characterization

Fifty-two NAFLD/NASH patients were categorized according to fibrosis severity: none, mild or advanced (Table 2). The mean age of the entire cohort was 52.1±10.5 years, 62% men. The three groups were matched for sex, age, and BMI. Liver function tests, glucose, and insulin were also similar among all three groups. However, triglycerides and platelet count were reduced in the advanced fibrosis cohort by ~34% (p<0.05) and ~27% (p<0.01) respectively, compared to the no fibrosis group. In turn, the non-invasive fibrosis panel FIB4, in which platelet count is a variable, was significantly increased with advanced fibrosis staging (Table 2).

**Table 2:** Subject characteristics.

Parameter	No Fibrosis (n = 20)	Mild Fibrosis (n = 20)	Advanced Fibrosis (n = 12)
Male (%)	65	55	67
Female (%)	35	45	33
Caucasian (%)	55	25	50
Hispanic (%)	30	55	42
African American (%)	5	5	8
Asian (%)	10	10	-
Filipino (%)	-	5	-
Age (years)	47.7±11.5	51.4±8.9	60.9±4.6
Weight (lbs)	214.6±40.4	212.1±48.6	210.3±55.0
BMI (kg/m^2^)	33.4±5.6	33.4±4.8	32.2±5.8
AST (U/L)	37.4±21.0	55.7±32.6	58.5±47.2
ALT (U/L)	60.1±36.8	84.2±47.7	62.0±46.9
Platelet count (x10^9^)	250.8±55.5	247.3±70.0**	183.5±115.5**^,^^##^
Triglyceride (mg/dL) †	159.8±85.5	172.7±124.5	105.3±26.4 *^,^^##^
LDL (mg/dL)	97.6±25.5	92.3±31.3	89.8±19.7
HDL (mg/dL)	40.4±12.6	46.5±13.3	44.1±14.5
Glucose (mg/dL) †	95.7±20.2	121.2±51.7	110.4±37.0
Insulin (uU/ml)	23.00±21.04	16.58±11.11	22.04±15.23
FIB4	1.19+0.56	1.57+0.64	2.97+1.64***^,^^###^
Liver Grade	0.50±0.51	1.45±0.51***	1.83±0.39***
Fibrosis Stages (%)			
F0	100	-	-
F1	-	50	-
F2	-	50	-
F3	-	-	83
F4	-	-	17

† n = 11 for advanced fibrosis cohort.

*p<0.05

** p<0.01

***p<0.001 vs No Fibrosis

##p<0.01

###p<0.001 vs Mild Fibrosis

### Inflammatory cytokines, macrophage and lymphocyte recruitment biomarkers

**Fig 2:**
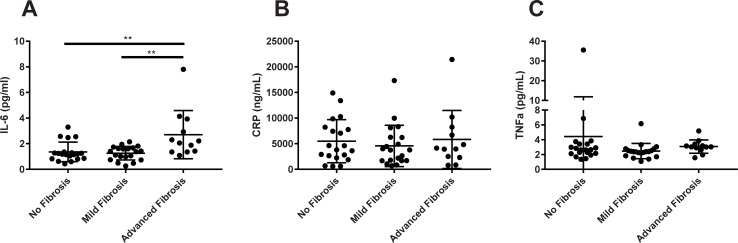
Inflammatory cytokines analyzed by NASH fibrosis severity. (A) IL-6, (B) CRP, and (C) TNF-α. **p<0.01 vs Advanced Fibrosis group.

Chronic inflammatory biomarkers such as IL-6, CRP and TNFα were measured in NAFL/NASH patients with varying degree of fibrosis. IL-6 was increased by 1.4-fold (p<0.01) in advanced fibrosis patients compared to patients with no fibrosis, and increased by 1.1-fold (p<0.01) compared to mild fibrosis patients (Fig 2A). IL-6 also demonstrated good performance as a biomarker of NASH fibrosis as demonstrated with an AUROC greater than 0.80 (Table 3). In contrast, no significant difference was observed between the groups for CRP (Fig 2B) nor TNFα (Fig 2C). Although one subject in the no fibrosis group displayed a TNFα value higher than their counterparts, this value is considered to be within physiological range [23].

**Table 3:** VCAM-1 outperforms the pro-inflammatory cytokines as a biomarker of no fibrosis versus advanced fibrosis.

Pro-inflammatory Cytokines	AUROC	95% CI	p-value
IL-6	0.83	0.67; 0.98	0.0024
CRP	0.51	0.30; 0.72	NS
TNFα	0.63	0.43; 0.83	NS
VCAM-1	0.87	0.75; 1.00	0.0005

AUROC, area under the receiver operating curve; CI, confidence intervals; NS, not statistically significant

**Fig 3:**
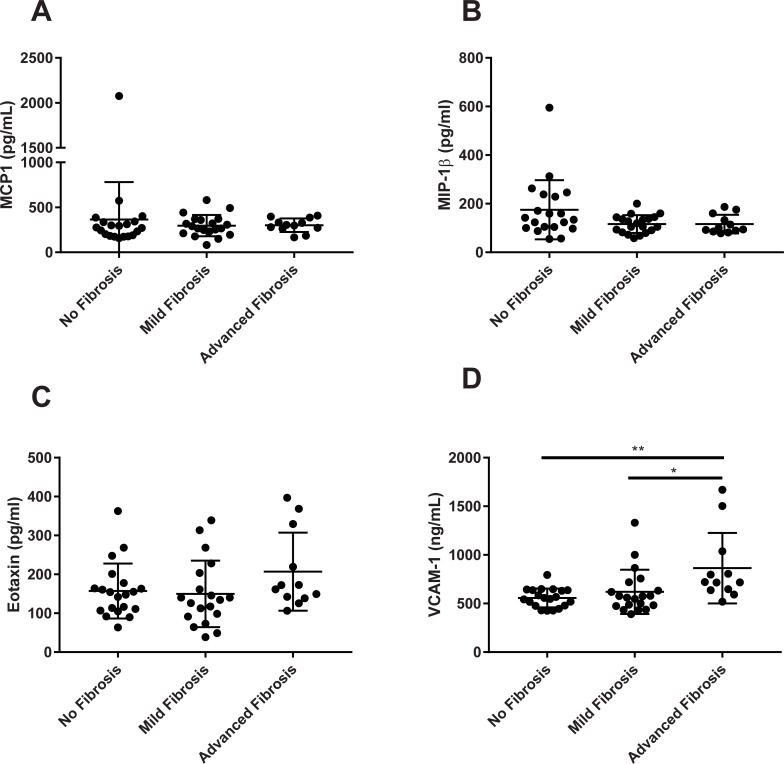
Chemokines and VCAM-1 analyzed by NASH fibrosis severity. (A) MCP-1, (B) MIP-1b, (C) Eotaxin and (D) VCAM-1. *p<0.05, **p<0.01 vs Advanced Fibrosis group.

**Fig 4:**
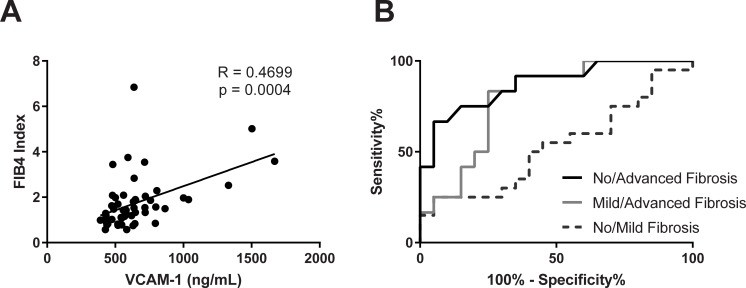
VCAM-1 demonstrates good performance in distinguishing advanced fibrosis in NASH. (A) VCAM-1 correlates with FIB4 values. (B) ROC curves demonstrating performance of VCAM-1 to distinguish fibrosis severity in NASH. AUROC of 0.87, 0.79, 0.53 for no/advanced, mild/advanced, and no/mild fibrosis severity respectively.

Biomarkers of macrophage recruitment such as MCP-1, MIP-1β, and eotaxin also demonstrated little change amongst the groups of fibrosis severity (Fig 3A–3C). On the other hand, VCAM-1 levels were elevated by 55% (p<0.01) and 40% (p<0.05) in the advanced and mild fibrosis groups compared to no fibrosis cohort, respectively (Fig 3D). Also, VCAM-1 positively correlated with FIB4 (Fig 4A). Furthermore, VCAM-1 demonstrated better performance to distinguish between no fibrosis from advanced stages (AUROC = 0.87; 95% CI = 0.75, 1.0; p = 0.0005) and mild fibrosis from advanced fibrosis (AUROC = 0.79; 95% CI = 0.63, 0.95; p = 0.0064). However, sensitivity was considered poor for distinguishing no fibrosis compared to mild fibrosis (AUROC = 0.53; 95% CI = 0.35, 0.71; p = NS) (Fig 4B). Based on AUROC values, VCAM-1 also outperformed the other cytokines as a NASH fibrosis biomarker (Table 3).

## Discussion

In the present study, a suite of pro-inflammatory cytokine and chemokine assays were analytically validated as well as evaluated in a clinical setting to demonstrate use as a biomarker for fibrosis severity in NASH patients. Analytical validation examines the performance of a test or assay. The level of validation depends on the assay COU and risk associated with the biomarker. All soluble biomarker assays in the present study were developed according to FDA guidelines [16] to be used as an exploratory endpoint for regulatory submission of a clinical trial, though additional tests can be performed for other COUs such as a secondary endpoint. Clinical performance is typically measured against a reference standard. For many NASH biomarkers, this analysis is against histological biopsied results. Here we demonstrated that IL-6 and VCAM-1 are increased in NASH patients with advanced fibrosis stages, and displayed good performance in distinguishing advanced fibrosis from milder stages.

IL-6 is expressed in the liver, upregulated with NASH and plasma IL-6 concentrations positively correlate with fibrosis stage in NASH [18]. Similarly, we demonstrated an increase in IL-6 with fibrosis severity. When combined as a biomarker panel in an algorithm; IL-6, adiponectin and cytokeratin 18 (CK18) displayed an AUROC of 0.90 with sensitivity and specificity of 85% and 86% respectively for discriminating fatty liver alone from NASH [24]. Carulli *et al*. found a polymorphism in the IL-6 gene (-174G/C) that was more prevalent in NASH versus NAFLD patients and associated with insulin resistance [25], potentially implicating IL-6 in the pathogenesis of the disease. Interestingly, others have shown a reduction in IL-6 levels during interventional studies, suggesting a role as a treatment response biomarker. In a small pilot trial, IL-6 levels were significantly reduced with lifestyle, and vitamin E supplementation in biopsy diagnosed NASH patients [26]. More recently, the effects of Rifaximin, a broad spectrum gut bacteria antibiotic, was examined in biopsy-proven NASH patients and found to reduce serum endotoxins as well as IL-6 concentrations [27]. Taken altogether, the results presented here and those observed by others support the development of IL-6 as a biomarker of NASH fibrosis and for treatment response evaluation.

VCAM-1 concentrations are elevated in the presence of liver diseases such as chronic hepatitis [28, 29] and cirrhosis [30]. Lefere *et al*. first demonstrated elevated VCAM-1 levels associated with hepatic fibrosis (≥F2) in a cohort of severely obese males undergoing bariatric surgery. While this patient population represents a portion of NASH subjects, the overall estimate of obesity prevalence among NASH patients is ~80% [31]. Further, the study authors did replicate and validate their results in a group of outpatient subjects from a hepatology clinic, a population more closely reflecting the participants in our study. Typically serum VCAM-1 ranges are approximately 340–1150 ng/mL in healthy adults [32], and we observed ranges from 391–1669 ng/mL in NAFLD/NASH patients. However, there is an order of magnitude difference between our study values and the Lefere results, which may be related to the measurement process, stressing the need for assay validation.

Nonetheless, the trend of increasing VCAM-1 with fibrosis severity is consistent between both studies. Indeed, similar to our findings, VCAM-1 displayed an AUROC of 0.80 for discriminating fibrosis stage 2 or greater and outperformed FIB4 as an indicator of advanced fibrosis in NASH [33]. VCAM-1 has been recognized as a good biomarker of NASH fibrosis by others as well. Yoshimura *et al*. performed a robust clinical examination of 261 biomolecules in 132 NASH patients. Diagnostic biomarkers of NASH fibrosis were determined based on data mining in a “factor module” scheme, where multiple mutually correlated results were considered as a single dataset. Within the factor module, VCAM-1 stood out as a biomarker of interest for NASH fibrosis and formed the basis of the FM-Fibro Index. In this [34] and a follow-up study [35], the FM-Fibro Index displayed diagnostic accuracy over 0.90 by AUROC when comparing mild (F0-2) to advanced (F3-4) fibrosis stages. On the other hand, in a recent large, multicenter study in biopsy-proven NASH patients, Itoh *et al*. found that FM-Fibro index had lower, although sufficient accuracy for predicting NASH-related fibrosis (AUROC ~0.70), yet excellent positive predictive value. Discrepancies between these studies included the number of patient with advanced fibrosis, as well as the number of pathologists and hepatologist reviewing the biopsy results [36]. Moreover, to date, three independent research groups, including ourselves, have illustrated the value of VCAM-1 as a biomarker of NASH fibrosis in adults. Interestingly, these findings seem to be related to adults only. In children and adolescents, VCAM-1 was elevated with obesity regardless of NAFLD diagnosis compared to age-matched lean controls [37]. Although fibrosis staging was not determined in the study, children can exhibit a distinct fibrosis pattern [38]. Therefore, the elevated VCAM-1 observed in children and adolescents may reflect the known role of VCAM-1 in obesity-induced endothelial dysfunction and angiogenesis rather than NASH fibrosis *per se*. Nevertheless, in an adult NAFLD/NASH population, VCAM-1 demonstrates robust performance as a fibrosis biomarker.

In contrast to the IL-6 and VCAM-1 results, CRP, TNFα, MCP-1, MIP-1β, and eotaxin levels did not change with fibrosis severity. CRP is a generic inflammatory biomarker and is increased with obesity and steatosis, but not NASH severity [39, 40]. Surprisingly, TNFα also did not differ with increasing fibrosis stages in the present study. TNFα is direct regulator VCAM-1 protein expression in a NFk-B dependent manner [13], and is upregulated in NAFLD patients [19, 41]. It is important to note that in the present study, VCAM-1 along with the other inflammatory cytokines were evaluated in a diseased population only. No age- and BMI- matched healthy control group was examined, as the no fibrosis cohort comprised of NAFLD and NASH patients. This may, in part, explain why we observed no change in TNFα levels. The mean TNFα concentration for a group of healthy 50–60 years old is reported to be 0.65 pg/mL, and this value increases with age [23]. Despite one outlier subject, the mean TNFα value we reported for a similar age range was at least 3 to 4 times higher in our NAFLD/NASH subjects. On the other hand, a large biomarker study of 648 well-characterized NAFLD patients found a strong association and higher TNFα levels with significant fibrosis when comparing stages F2-F4 vs. F0-F1 [42]. Therefore, our sample size may not have been robust enough to detect a difference in TNFα according to fibrosis severity.

Interestingly, MCP-1 gene expression is upregulated in human liver tissue sampled from NAFL patients [43]. Haukeland *et al*. demonstrated that patients with NAFLD have a low-grade systemic inflammation and presented with higher serum levels of MCP-1 compared to controls [44]. In addition, MCP-1 levels were higher in NAFLD children with severe fibrosis [45]. MCP-1 binds acts via the C-C chemokine receptor 2 (CCR2) to promote monocyte and macrophage recruitment, migration and infiltration. Cenicriviroc, a dual CCR2/CCR5 antagonist demonstrated anti-fibrotic and anti-inflammation properties in a NASH clinical study [46]. However, similar to results presented here, Page *et al*. also found no significant change in MCP-1 concentrations with varying degrees of liver fibrosis in a systems-biology based approach to NASH biomarker identification [47]. To our knowledge, this is the first study to examine the relationship of NASH fibrosis and serum expression of MIP-1β and eotaxin. Both these chemokines were previously measured in a cohort of ultrasound diagnosed NAFL obese patients, and while eotaxin was associated with measures of insulin resistance (e.g. HOMA) and pro-inflammatory cytokines, it was not associated with ultrasound liver fat [48].

Incorporating inflammation and fibrosis specific factors into biomarker panels can increase performance and sensitivity. Clinical predictors of advanced fibrosis in NASH patients using innovative biomarker combinations such as FIB-C3 (pro-C3, age, BMI, diabetes, and platelet); FIBROSpect test (α2-macroglobulin, hyaluronic acid (HA), tissue inhibitor of metalloproteinase-1 (TIMP-1)); and HA+CK18+TIMP-1 yielded AUROC of 0.86, 0.87, 0.90 respectively (reviewed in [49]). Interestingly, the clinical performance of these panels is similar to the AUROC obtained for IL-6 and VCAM-1. Our study supports the belief that the addition of biomarkers such as IL-6 and VCAM-1 to panels such as FIB4 may yield increased sensitivity and specificity.

There are a number of study limitations that must be addressed. Due to the small sample size, patients were categorized into three groups (no, mild or advanced fibrosis), rather than evaluate each fibrosis stage separately. This approach was taken since only two patients were identified as F4 cirrhosis. Another limitation is that we only examined the biomarkers in a “test cohort”, and did not repeat the analysis in a separate “validation cohort”. Finally, due to the prospective nature of the study, we cannot evaluate a causal relationship between IL-6 and VCAM-1 and NASH fibrosis progression.

## Conclusion

In a single study comparison, the role of several cytokines and chemokines as biomarkers for NASH fibrosis were examined. IL-6 and VCAM-1 are a potential soluble biomarker for NASH risk stratification, able to discriminate between mild and severe stages of fibrosis. Moreover, VCAM-1 outperforms the pro-inflammatory cytokines and chemokines examined in the present study. Analytical validation is also required to satisfy regulatory guidance for biomarker use in a clinical trial. To this end, we demonstrated good accuracy and assay robustness for VCAM-1 as well as clinical value.

## Abbreviations

COU: context of use
CRP: C-reactive protein
HSC: hepatic stellate cell
IL-6: inteulin-6
NAFLD: nonalcoholic fatty liver disease
MCP-1: macrophage chemoattractant protein 1
MIP1β: macrophage inflammation protein 1 beta
NASH: nonalcoholic steatohepatitis
TNFα: tumor necrosis factor alpha
VCAM-1: vascular cell adhesion molecule 1
